# Virile Infertile Men, and Other Representations of In/Fertile Hegemonic Masculinity in Fiction Television Series

**DOI:** 10.1007/s10912-020-09647-1

**Published:** 2020-09-25

**Authors:** Marjolein Lotte de Boer

**Affiliations:** Department of Culture Studies, Tilburg School of Humanities & Digital Sciences, Tilburg University, P.O. Box 90153 5000, LE Tilburg, The Netherlands

**Keywords:** Male infertility, Hegemonic masculinity, Power, Ricoeur, Representations, Fiction television series, Content analysis

## Abstract

Fiction television series are one of the few cultural expressions in which men’s infertility experiences are represented. Through a content analysis of twenty fiction series, this article describes and analyzes such representations. By drawing on Connell’s concept of hegemonic masculinity and Ricoeur’s understanding of paradoxical power structuring, four character types of infertile men are identified: (1) the virile in/fertile man, (2) the secretly non-/vasectomized man, (3) the intellectual eunuch, (4) the enslaving post-apocalyptic man. While these various dramatis persona outline different ways of how infertile men relate to normative hegemonic masculinity, they all represent infertile men as diverging from shared masculine norms. This non-normativity initially excludes many represented men from hegemonic positions. Eventually, however, these men generally aspire to and succeed in reaffirming their hegemonic masculinity through coercive force towards women and other men, through instigating the precondition for any power structure – the shared will to live together as a community –, and/or by seeking and finding explicit recognition for their normativity and dominance. At the end of this paper, I will reflect on the potential harmful effect of these outlined representations of infertile men and make a plea for diversifying representations of infertile men in our culture.

## Introduction

Male infertility is not an uncommon phenomenon. Approximately 40% of couples’ infertility can be traced back to the man (Agarwal et al. 2015). Moreover, an estimated 2.4% of men within couples who do not want to have more children have a vasectomy through which they become voluntarily infertile (Shattuck et al. 2016). Given the large number of men who experience and deal with infertility, it is surprising how sporadic representations of male infertility experiences are in our Western culture. In public life, infertility is predominantly framed as a female issue (De Boer, Archetti, and Solbraekke 2019; Edge 2015). While some media occasionally represent male infertility, these cultural expressions typically reduce the phenomenon to anthropomorphic portrayals of “unmotivated,” “feminizing” (Gannon, Glover, and Abel 2004, 1173), “non-romantic,” “slow,” “sad,” and even “loser” (Moore, 2003, 289-290) sperm cells that are not able to fertilize an egg cell. Arguably, one of the few contemporary cultural expressions that frequently represent actual men who deal with their infertility is fiction television series. This study offers an analysis of ‘male infertility fiction series’ and the representations of men’s infertility experiences. In doing so, I explore the meaning of masculinity in our culture. How are men’s infertility experiences represented in fiction television series? What do these representations reveal about the ways in which we perceive and deal with masculinity in our culture?

## The significance of male infertility representations

Describing and analyzing representations of male infertility experiences offers an opportunity to gain insight into what masculinity means in everyday life (Gannon, Glover, and Abel 2004). Todd Reeser (2011) argues that as a default position, masculinity is often a taken-for-granted aspect of life. It is something that passes by invisibly and only flickers into people’s attention “when something goes wrong or when it goes into excessive overdrive” (1). Representations of men’s lived infertility may be understood as examples of masculinity ‘going wrong.’ That is, their infertility may undercut the traditional equation of accepted or normative masculinity with virility (Cragun and Sumerau 2017; Mason 2003; Thompson 2005) or with the (biological) father role (Nachtigall, Becker, and Wozny 1992; Owens 1982; Schmitz 2016). In falling short of the norm, then, representations of men who experience and deal with their infertility may help to draw out how variants of masculinity are at play in our culture.

Moreover, besides providing insight into cultural perceptions of masculinity, this study pertains to the very basis of existence: to (male) identity construction. The hermeneutical philosopher Paul Ricoeur (1991) teaches us that we make sense of ourselves through telling and re-telling stories about ourselves during which we continually refer to larger stories that roam around in our culture. This means that in our stories about who we are, we relate to characters in cultural narratives: we recognize, compare, contrast, match, and connect with them in order to constitute a sense of ourselves. In this sense, representations of others in cultural expressions are the make-up of our identities: they are the basis for and precondition of selves’ sense-making structures. Representations of men’s infertility experiences in fiction television series, then, provide a window into understanding how cultural perceptions of infertility and masculinity may have real repercussions for the ways in which in/fertile men are un/able to make sense of themselves.

Despite the importance of studying representations of male infertility, systematic empirical research of these representations is scarce. Some studies examine representations of sperm in popular culture and in doing so, reveal interesting aspects of perceptions of non-/normative masculinity. They show that infertile sperm cells – in being branded as “feminizing” (Gannon, Glover, and Abel 2004, 1173), “slow,” or “loser[s]” (Moore 2003, 289-290) – are characterized as being unable to live up to norms of masculinity. These studies then typically proceed by suggesting that the characterizations of biological material reflect and co-construct cultural understandings of infertile men. However, by merely studying representations of infertile sperm cells, such studies offer rather indirect and suggestive insights into cultural perceptions of infertile men and masculinity and what such perceptions may mean for men’s identity construction. Therefore, by turning our attention to describing and analyzing representations of men living with and through their infertility, in this case in fiction television series, this study aims to offer a more comprehensive understanding of represented in/fertile masculinity.

## Theoretical framework: hegemonic masculinity

To study representations of men’s infertility experiences, I employ the concept of hegemonic masculinity as a theoretical guideline, which became well-known through the works of masculinity studies theorist, Robert Connell (2005; Connell and Messerschmidt 2005), and reminds us that masculinity relates to power structures. Connell (2005) contends that in our Western culture, men are encouraged to enact and subscribe to a set of social norms in order to be legitimized as being in a position of power, that is, in a position of hegemony over others (Stern and Buikema 2013). It is argued that some of these norms explicitly relate to male fertility, that is, to men’s ability to make women pregnant (Cragun and Sumerau 2017), to the role of men as the head of and provider for their (biological) families, to men having offspring and an heir (Schmitz 2016), or to men’s ability to have an erection and to ejaculate (Clarke, Marks, and Lykins 2015; Johnson Jr 2010). Moreover, norms that legitimize men’s powerful positions may also adhere to other lived features like being strong (Wetherell and Edley 1999), tall (Talbot and Quayle 2010), rational (Bird 1996; Wetherell and Edley 1999), or emotionally stoic (Greenebaum and Dexter 2018). These latter norms, in turn, may intersect with masculine norms related to in/fertility. For example, virile men may be expected to live up to norms of rationality and strength as well (Greenebaum and Dexter 2018; Johnson Jr 2010). In taking the concept of hegemonic masculinity as a heuristic tool, then, this study teases out how representations of men living with their infertility relate to certain masculine norms, and, by extension, whether and how these represented men may be understood as being in a position of power. Before expanding on my empirical results, however, let me elaborate on my theoretical understanding of hegemonic masculinity.

Central to the concept of hegemonic masculinity is the aspect of relationality, the concept that refers to normative structures legitimizing and enabling men to dominate others (Connell 2005; Jewkes and Morrell 2010). Moreover, it is argued that living up to these hegemonic norms is typically not a given quality but rather a matter of aspiration; thus hegemonic masculinity entails constant negotiations with others (Connell 2005; Shefer, Ratele, and Strebel 2007). That is, men have to continually procure, argue for, or defend their normativity and dominance in relation to others. It is significant to note here that within and through these negotiations, positions of power, as well as the norms of masculinity underlying these positions, may change (Connell and Messerschmidt 2005). In appreciating this pivotal aspect of relationality and changeability in hegemonic masculinity, I therefore assess how normative, hegemonic masculinity is negotiated in representations of infertile men in fiction television series and how such negotiations may possibly result in power shifts and/or altered understandings of masculinity within these series.

In analyzing such relational hegemonic masculinity in male infertility fiction television series, it seems constructive to look beyond masculinity studies. While several masculinity studies scholars argue for acknowledging the significance of relationality and negotiation in hegemonic masculinity (Brod and Kaufman 1994; Connell and Messerschmidt 2005; Talbot and Quayle 2010), this field of research grapples with restrictive interpretations of relationality.

First of all, empirical analyses of negotiated hegemonic masculinity within (but also beyond) the context of male infertility experiences predominantly examine relationships between men: they show how men amongst each other understand and negotiate (their) normativity and hegemony (Bird 1996; Connell and Messerschmidt 2005; Diffrient 2017; Dolan et al. 2017; Lee and Chu 2001; Malik and Coulson 2008; Mason 2003; Webb and Daniluk 1999). In doing so, these studies position women at the periphery of constructions of masculine norms and hegemonic masculinity, rather than understanding them as significant actors in the negotiation of hegemonic masculinity (Brod and Kaufman 1994; Connell and Messerschmidt 2005). By drawing on relevant feminist and anthropological studies, I aim to be attentive to the larger gendered dimension within the negotiation of normative, hegemonic masculinity in the context of male infertility. As we will see, these studies help to demonstrate how representations of male infertility experiences are shaped and co-constituted within and through, for example, relational dynamics between men and women and/or (shared) normative ideas about how power(lessness) is inscribed in gendered bodies (Askey 2018; Gjelsvik and Schubart 2016; Inhorn 2003; Scholz 2001; Tougher 2002; Schilt 2006). Moreover, these studies help to understand that ritualizing certain well-known cultural stories may establish and cultivate a tradition of female subordination and enslavement (Kitts 2010; Tambiah 1979).

Second, as many masculinity studies scholars (Bird 1996; Groes-Green 2009; Hunter, Riggs, and Augoustinos 2017; Mani 2018; Morrell, Kewkes, and Lindegger 2012) interpret the concept of hegemony and its relational aspect along the lines of Connell (2005), it may be argued that while their understanding of relational power is insightful, it is also rather restricted. Connell, as well as his successors view power as something that is acquired and legitimatized through processes of consent and complicity by the ruled and through persuasion and coercive force by the ruling (Bird 1996; Connell 2005; Connell and Messerschmidt 2005; Groes-Green 2009; Hunter, Riggs, and Augoustinos 2017). In my view, Ricoeur (2000) offers in his political philosophical works a welcome contribution to this understanding of negotiation and power by showing that power structuring cannot simply rest on consent and/or coercion but rather is inherently enigmatic and paradoxical. As such, Ricoeur’s thinking together with that of Connell serves as a fitting tool in invigorating ways of analyzing negotiations of masculine norms and hegemonic masculinity in representations of men dealing with their infertility in fiction television series.

In his works on power, Ricoeur (2000, 2007) shows that those who (aim to) acquire or defend a hegemonic position need to also look beyond a persuasion/coercion toolbox. For Ricoeur, to be persuaded means to actually believe in what one is persuaded to do or to think. This implies that persuasion occurs through a (basic) set of already shared ideas that bring together (ruled) audiences and (ruling) rhetors. Such communality functions as a ground of premises that enable the audience to arrive, through argumentation, at the same conclusions as the rhetors. So while persuasion does not rest on absolute equality and symmetry – there is always a party who persuades and one who is persuaded – it presupposes a large degree of equality and symmetry much as in as persuasion presupposes a set of shared ideas, works through a process of argumentation, and leads to arriving at a common conclusion. As such, power cannot rest on persuasion alone in as much as the former may conserve a notion of (great) hierarchical difference and ideological dissymmetry between those in power and those who are not. Power, according to Ricoeur, cannot rest solely on coercion either. For him, the origin of power lies in the will to live together as a group or community. Power, he writes, “only exists as long as this (…) will is effective” (2000, 8). If this will would not be in place, people would disperse in mere plurality. By implication, then, power cannot be gained through or maintained with coercion alone, as violence may ultimately undermine the very basis for power: the will to live together. What is required in attaining and maintaining a hegemonic position, then, is recognition. It is, Ricoeur (2000) argues, “between the demand for recognition of (the ruling) and the capacity for recognition on the part of the subordinate parties that the ruling parties are able to command, and in doing so, be obeyed” (22). But if hegemony or power is not accredited solely through persuasion or coercion but is also authorized by (seeking) recognition, where does this recognition come from? How, in other words, is power authorized?

Here, Ricoeur (2000) develops his idea of – what he calls – the ‘paradox’ and ‘enigma’ of power. He contends that power is ultimately authorized within and through the mediation of a higher power, like a government or monarch, who legitimizes the distinction of the ruling and the ruled, that is, the hierarchical instance upon which power rests. But as the question ‘what authorizes their power?’ remains – upon which a new reference to an even higher power is required – authority eventually “comes from elsewhere, far away and far higher than the power itself” (7). An example of such a “far away” authority is a religious tradition and its ecclesiastical teachings. However, be it the Christian, Islamic, or Buddhist teachings, their professed power structures ultimately also rest on a belief in the authority of their authority. What these stories of origin and historical traditions offer us, then, is not a definite foundation for power but a tradition of faith in the trustworthiness of the authority of the power structure they profess. At this point, Ricoeur (2007) reveals power structures as both enigmatic and paradoxical. “Whoever we are,” he writes, be it “subordinates or in charge, we do not really know what authorized authority” (94). Through this foundational enigma at the heart of power, then, it becomes clear that power is paradoxical: while it is dependent on people’s will to recognize and believe in its legitimacy, it is ultimately self-referential: “the ‘people’ authorize itself” (94).

This understanding of enigmatic, paradoxical power structuring, together with Connell’s understanding of power as a matter of persuasion and coercion, may help to understand negotiations of masculine norms and hegemonic masculinity in representations of men dealing with their infertility. In this study, therefore, we specifically ask whether and how the represented in/fertile men establish, defend, and remain in a position of power by way of persuading, forcing, and/or by seeking recognition with other men and women.

## Methods: researching representations of in/fertile masculinity

By way of a content analysis, this study identifies and analyzes whether and how representations of infertile men in fiction television series include negotiations of masculine norms and hegemonic masculinity. For the purpose of this study, I collected and analyzed twenty English-speaking series in which men’s infertility experiences is a significant plotline, meaning that the selected series portray men dealing with their infertility in multiple scenes in one episode and/or throughout multiple episodes in one or more seasons. The study’s sample includes mostly sitcoms such as *How I Met Your Mother* and *Modern Family* but also historical fiction series like *Downton Abbey* as well as science fiction series such as *Game of Thrones*, *The Handmaid’s Tail* and *The Lottery*. Since this study deals with contemporary representations of men’s infertility experiences, most of these television series first aired in the last decade and many of them are still running. However, I also included fiction television series that date back to the 1990s (*Friends, Sopranos, Home Improvement*) that are still readily available on on-demand television and Internet channels such as Netflix, HBO, and YouTube. Because of their current availability on popular television and Internet channels, the representations of men’s infertility experiences arguably still actively contribute to contemporary cultural perceptions of masculinity. See Table 1 for more information about the sampled shows.

**Table 1:** Details selected series

Identified *Dramatis persona*	Series	Channel/ Year	Season/ Episodes	Infertile male character
*The virile in/fertile man*	How I Met Your Mother	CBS (2005 – 2014)	S6 E12, E13	Marshall
	Tell Me You Love Me	HBO (2007)	S1 E2, E3, E4, E5	Patel
	Downton Abbey	ITV/PBS (2010 – 2015)	S2 E5, E6, E7, E8	Matthew
	Friends	NBC (1994 – 2004)	S9 E21, 22; S10 E9	Chandler
	Masters of Sex	Showtime (2013 – 2016)	S1 E1	Bill
	You Me Her	Netflix (2016 – present)	S1, all episodes	Jack
*The secretly non-/vasectomized man*	Californication	Showtime (2007 – 2014)	S2 E1	Hank
	Modern Family	ABC (2009 – present)	S4 E4	Phil
	The Sopranos	HBO (1999 – 2007)	S2E9	Tony
	Gilmore Girls	The WB (2000 – 2007)	S7 E12, E21	Jackson
	About a Boy	NBC (2014 – 2015)	S2E1	Andy
	Home Improvement	ABC (1991 – 1999)	S5, E16	Tim
	Married	FX (2014 – 2015)	S1 E6	Russ
	Black-ish	ABC (2014 – present)	S1E18	Dre
	Reba	The WB (2001 – 2007)	S2 E12	Brock
	Real Rob	Netflix (2015 – present)	S1 E2, E3	Rob
*The intellectual eunuch*	Vikings	History Channel (2013 – present)	S4, E1	Einar
	Game of Thrones	HBO (2011 – present)	S1, 2, 3, 4, 5, 6, 7 (all episodes)	Lord Varys, Grey Worm, Theon Greyjoy
*The enslaving post-apocalyptic man*	The Lottery	Lifetime (2014)	S1	Kyle
	The Handmaid’s Tale	Hulu (2017 – present)	S1	Commander Fred Waterford

In the analysis phase, I watched and re-watched the collected episodes and/or seasons, transcribed conversations and monologues wherein male infertility is discussed and dealt with, and made notes about how men’s infertility experiences are portrayed and verbalized through the scene’s setting, the actors’ gestures, their facial expressions, and their vocal use. By drawing on relevant theories about variants of (normative, hegemonic) masculinity in our culture (Gannon, Glover, and Abel 2004; Cragun and Sumerau 2017; Greenebaum and Dexter 2018; Inhorn 2003; Schilt 2006; Schmitz 2016; Scholz 2001), the transcriptions of relevant excerpts of the series, and the extensive notes I took while watching (and re-watching) these series, I identified recurring themes and sub-plots related to infertile men’s negotiations with (their) masculinity. An example of such a theme is ‘Questioning hegemonic masculinity: virility,’ which includes all portrayals of (alleged) infertile men whose hegemonic masculinity was questioned because they were not (considered) manly enough (see particularly the character type of ‘the virile infertile man’). Two other examples of such themes are: ‘Reaffirmed hegemonic masculinity: enslaving women’ and ‘Reaffirmed hegemonic masculinity: intellectual supremacy.’ These two themes include all portrayals of (alleged) infertile men reaffirming their hegemonic position by enslaving women (see particularly the character type of ‘the enslaving post-apocalyptic man’) or through displaying their intellectual supremacy (see particularly the character type of ‘the intellectual eunuch’). By coding and re-coding these different themes, thereby identifying and analyzing their interconnected structures, this analysis eventually resulted in the identification of four different character types or dramatis persona of infertile men who negotiate with normative, hegemonic masculinity: ‘the virile in/fertile man,’ ‘the secretly non-/vasectomized man,’ ‘the intellectual eunuch,’ and as ‘the enslaving post-apocalyptic man.’

## Results: four representations of in/fertile men

Every represented infertile man in the four identified dramatis persona is confronted with questioning his hegemonic masculinity through accentuating and exaggerating divergence from and failure to live up to various masculine norms – e.g. norms of virility, having offspring, emotional stoicism, physical strength, or having muscular bodily forms. Generally, these men are eventually able to live up to norms of masculinity and take up hegemonic positions. This quest to (re)instate their hegemony often goes together with undermining and forcefully subverting other men and women and/or with instigating the precondition for any power structure – the shared will to live together as a community –, as well as with seeking and finding recognition for their normativity and dominance. That is, in the series that portray men who are possibly involuntarily infertile and have fertility tests, their hegemony is generally reaffirmed through test results that confirm fertility and/or through their proven ability to make their partners pregnant. But even when such a test result is negative and a man is represented as actually involuntary infertile, his hegemonic position is paradoxically still affirmed through his acknowledged ability to live up to the norm of being a providing father and a virile man (see: the virile in/fertile man). In other series, some men consider having a vasectomy in response to their (female) partners’ wishes or demands, upon which their normative masculinity and hegemonic position is severely questioned. Their hegemonic masculinity, however, is typically reinstated within and through their choice of secretly not having a vasectomy. This choice allows men to (continue to) be a normative, virile man. Moreover, as this choice results in an unexpected, male imposed and eventually celebrated pregnancy, it also fosters an environment in which the female, subjugated partners explicate their will to live together within a male-dominated family (see: the secretly non-/vasectomized man). In yet other series, castrated men’s questioning of hegemonic masculinity is restored through refocusing from their bodily, phallocentric inferiority to their intellectual supremacy (see: the intellectual eunuch). Finally, in two other series where male as well as female infertility threatens the survival of humankind, the hegemonic positions of the supposedly infertile men are confirmed by their eventual ability to procreate by violently enslaving women for reproductive purposes and by cultivating a tradition of faith in the legitimacy of men’s domination (see: the enslaving post-apocalyptic man). Besides from these plotlines of reinstated in/fertile hegemonic masculinity, I will show that some fiction television series also occasionally depict non-hegemonic or ambiguously non-/hegemonic in/fertile masculinities.

## The virile in/fertile man

Fiction television series that focus on couples’ inability to conceive include men’s surmise that they might be infertile and – when the series takes place in the present – should get a fertility test. In these series, men’s pending in/fertility goes along with questioning their virile and potent hegemonic masculinity, which is expressed in either their self-hatred or their shame towards women or other (more) hegemonic males. For instance, when hearing that he might be infertile, Matthew in *Downton Abbey* says that “no one would want to be with me (…), [being] impotent, stinking.” Marshall in *How I Met Your Mother,* moreover, refrains from telling his stereotypical masculine father – who is only portrayed while fixing things and drinking beer – about his fertility test, even though Marshall states that they “share everything.”

Interestingly, the storyline of these men’s uncertain hegemony does not develop with them holding a subversive position but rather becoming a powerful reaffirmation of their hegemonic masculinity. In most series, this reaffirmation takes place through the dynamic of depicting these men as living up to certain norms of hegemonic masculinity males whilst portraying their female partners as subordinate. In *How I Met Your Mother*, this kind of hegemonic reaffirmation dynamic takes a rather absurd turn. While Marshall is in the bathroom trying to masturbate in order to provide a semen sample for his fertility test, his partner, Lily, receives his parents at the door. As they greet each other, Marshall’s mother asks the strange question whether Lily has become shorter since the last time they saw each other (i.e. a couple of weeks ago). Since Lily and Marshall’s height difference – Lily is much shorter than Marshall – is a running gag in the show, it may be argued that by augmenting Lily’s shortness, Marshall’s tallness – especially in relation to Lily – is accentuated through which his hegemonic masculinity is reaffirmed. After all, the legitimation of hegemonic masculinity may take place through adhering to lived features such as men’s tallness (Talbot and Quayle 2010). That is, tall male bodies are traditionally understood as a signifier of hegemonic traits such as leadership, dominance, and the entitlement to take up actual and symbolic space, whereas short female bodies invoke powerlessness and spatial modesty (Schilt 2006).

While most series eventually include the ultimate reaffirmation of these men’s hegemonic masculinity by portraying them as fertile, two series depict men’s continued infertility and their inability to have biological children. In *Masters of Sex*, Bill’s infertility seems to be merely used as a way to explain his interest in researching sexuality, but in *Friends*, Chandler’s infertility is a major plotline throughout a whole season. Given that the men’s infertility already appears to be a major assault on their hegemonic masculinity, we may expect that with Chandler’s persistent infertility, he is deemed for a subordinate position. However, at the same time that Chandler is indeed portrayed as a non-hegemonic male – up to a point that Chandler suggests that his partner, Monica, should have a baby with a “better man” – his hegemony is also reaffirmed. A key aspect in understanding Chandler’s ambiguous infertile masculinity is the appurtenant narrative that Monica is also infertile, and the way in which their shared infertility is narrated. A telling moment in this narration is when Chandler, who has just hung up the phone with their fertility doctor, breaks the news of their shared infertility to Monica. He says:

Apparently, my sperm has low motility, and you have an inhospitable environment. […] It means that my guys do not get out of their Barcaloungers, and you have a uterus that is prepared to kill the ones that do. (*Friends*, season 9, episode 21)

What stands out in this quote is the metaphor that is used in order to describe Chandler’s and Monica’s shared infertility. It may be interpreted – similar to studies arguing that anthropomorphizing descriptions of infertile sperm cells reflect perceptions of non-normative masculinity (Gannon, Glover, and Abel 2004; Moore 2003) – that Chandler’s “low motility” sperm cells echoes his non-normative, inactive, and docile masculinity (Trautner, Kwan, and Savage 2013).

However, within this same quote, Chandler’s normative and hegemonic masculinity also seems to be affirmed, albeit ambiguously. That is, Chandler’s ambivalent masculinity seems to take shape along the lines of – what Inhorn (2003) calls – one of the “patriarchal paradox[es] of [hegemonic] male procreation” (246). This paradox entails that while male infertility seems to conflict with the hegemonic ideal of virility, potency, and procreation, men’s infertility – especially when they are able to ejaculate and as presented in tandem with female infertility – does not actually redound on this ideal. For Chandler this means that as his words reveal that he is still able pass (some of) his ejaculated sperm into Monica’s uterus, he, even in his alleged infertility and his inability to conceive biological children with his partner, is represented as a potentially reproductive male. It is, after all, Monica who is presented as ultimately barring procreation (and Chandler’s efforts herein) by “killing” his sperm cells that do “get out of their Barcaloungers” and into her uterus. The combination of Chandler’s ejaculating capacities and Monica’s infertility that bars their reproduction, then, deems Chandler in the paradoxical position of always living up to the masculine norm of being a virile and (potentially) procreative man, even when he is practically infertile (Inhorn 2003).

Interestingly, in the next season, when he and Monica decide to adopt a child, Chandler is dislodged from his masculine ambivalence and explicitly recognized as a hegemonic male. Herein, a pivotal plot line is that during the process of becoming adoptive parents, the couple lie about who they are to the potential birth mother. Chandler pretends to be a medical doctor and Monica a minister. In an ironic plot twist, Chandler – in contrast to Monica – wants to do “the good thing” and come clean to their potential birth mother. Upon hearing the truth, the birth mother initially does not want to give the couple her child anymore, but Chandler is eventually able to persuade her otherwise. From then on, Chandler’s normative, hegemonic masculinity is no longer questioned in the series. It seems that within his quest to become an adoptive parent, Chandler lives up to certain norms of masculinity. That is, by arranging and securing the essence of their desired family life – a child – in an honest way, he seems to live up to the masculine norm of not only the providing father – quite literally by providing a child – but also to the ideal of the hero with civic virtues (Fleras and Dixon 2012; Gardiner 2002; Holt and Thompson 2004).

Within this plot line, moreover, Chandler is not only presented as a normative, hegemonic male, he also seems to be recognized as such by Monica. When Chandler tells Monica that he was able to persuade the birth mother to give them her child, she says: “God bless you, Chandler.” It may be argued that here we see Ricoeur’s (2000) theory of recognizing power in practice, a theory which states that the recognition of power within a community is a pivotal aspect in establishing and authorizing power structures. This theory further contends that since we cannot provide a definite foundation for authorizing power structures, such an authorization eventually “comes from elsewhere, far away and far higher than the power itself” (Ricoeur 2000, 7). In the case of *Friends*, then, Chandler’s paternal and virtuous masculinity is explicitly acknowledged with a comic nod to his and Monica’s identity lies. That is, Monica seems to recognize Chandler’s normative hegemonic masculinity within and through her ‘authority’ as a pretend-minister to bless him for being that way in the face of a higher religious power.

## The secretly non-/vasectomized man

Besides unwanted infertility, a few fiction television series include a narrative of voluntary male sterilization, namely when a man has or considers having a vasectomy. Such narratives generally involve the assumption that men who have a vasectomy are not ‘real’ men, something which seems to implicate an inability to live up to hegemonic masculine norms of bodily and emotional toughness (Greenebaum and Dexter 2018). For example, when Phil in *Modern Family* sits on a bench pondering whether he will have a vasectomy, the text on the back of the bench reads: “not a real man.” All the while, he talks about his anxieties to his father-in-law, Jay – the patriarch of the family – and confesses that he is “afraid it [the vasectomy] will hurt,” as he is “unusually sensitive down there.” At the end of the scene, Phil’s confession of his sensitive nature results in being subordinated when Jay compares Phil with “any other little girl.”

Given that fiction television series represent vasectomized men as not ‘real’ and non-hegemonic, it is hardly surprising that the represented men rarely initiate having a vasectomy. Rather, these men only consider having a vasectomy after their female partners ask or even demand that they have one. Eventually, however, almost all of these men rebel against their partners’ wishes or demands by secretly not having a vasectomy. As a vasectomy is framed as losing one’s ‘real’ or normative hegemonic masculinity, such deceitful rebellion can be understood as a reaffirmation of hegemony. That is, by not having a vasectomy, these men not only continue to live up to the norm of virile masculinity (Cragun and Sumerau 2017; Johnson Jr 2010), but by disobeying their female partners, they also avert a subservient position in relation to them.

Moreover, in secretly not having a vasectomy, these men implicitly subject female partners to their reproductive choice; their deceit is unmasked with a positive pregnancy test. In the context of this unwanted, arguably enforced pregnancy, such men are presented as “stupid” (*Gilmore Girls*), “fools” (*About a Boy*) and “bastards” (*Blask-ish*) for making unilateral reproductive choices. At the same time, however, their actions are also presented as understandable and ultimately unproblematic. For example, when Jackson in *Gilmore Girls* tells Lorelai, his partner’s best friend, that he did not have a vasectomy, Lorelai says he is “stupid” but also that she understands that “you don’t get a vasectomy just because someone [in this case, his partner] tells you to.” Then, when his partner, Sooki, is upset after finding out she is pregnant again, Lorelai tries to get Sooki excited for having another baby. Lorelai seems to succeed in doing so as the episode ends with Sooki enthusiastically talking about the wonders of having a newborn. It may be interpreted that through this eventually celebrated pregnant outcome of Jackson’s rebellion, his hegemonic position is affirmed. Welcoming a new life into a hierarchical, male hegemonic family may be understood as an appearance of what Ricoeur (2000) claims to be the foundation of power: the will to (continue to) live together, in this case, within a hierarchical family. In this sense, Sooki’s celebration of her pregnancy seems to convey a recognition for her partner’s hegemonic position through the affirmative to continue to live with him.

Note that, interestingly, one television series does not follow the plotline of men secretly not having a vasectomy and women becoming unexpectedly pregnant. In the show *Reba*, Brock secretly had a vasectomy after having his first baby with his wife, Barbra-Jean. When Barbra-Jean finds out Brock had a secret vasectomy, she demands he have the vasectomy reversed. Eventually, he reluctantly complies. At first sight, the plot of reversing a vasectomy seems to underline, just as in the abovementioned series, that normative masculinity implicates living up to the norm of virility. However, in contrast to the other accounted vasectomy series, Brock’s portrayed virility also seems to challenge his normative, hegemonic masculinity. After all, he lives up (again) to the norm of virility in reluctantly complying with his wife’s demand of reversing his vasectomy. It could be argued, then, that in being a virile man, Brock not only attests to normative masculinity but is also subservient to his wife.

## The intellectual eunuch

Castrated male characters or so-called ‘eunuchs’ are occasionally part of fiction television series that take place in medieval-inspired worlds. In such series, these men’s genitals are generally involuntarily removed for political reasons: as a form of punishment or as part of disciplining practices. Out of the analyzed television series in which eunuchs play a major role, *Game of Thrones* is particularly interesting. This series includes three major eunuch characters: Theon Greyjoy, Lord Varys, and Grey Worm. What these eunuchs have in common is that they seem to fall outside of the heteronormative phallocentric masculine norm because of their lack of male genitals. In *Game of Thrones*’ societies, the penis is often displayed as a heterosexual symbol of male dominance. Men regularly dominate women by means of penetration, or they flash their (urinating) penis to impress, scare, or humiliate other men and women. It would seem that the eunuchs’ lack of male genitals deprives them of such a position of phallic power.

This kind of power deprivation is indeed the case for Theon Greyjoy. In his pre-eunuch life, Theon Greyjoy displayed heterosexual phallocentrism in repeatedly dominating women during sex. This hegemonic position is terminated when he is castrated as part of his tortuous captivity. Post-castration, Theon Greyjoy becomes an obedient servant to his torturer who repeatedly makes an effort to prove that his castrated slave is unable to dominate women through his penis. What is more, Theon Greyjoy also seems to be stripped from his personhood upon castration. After his genitals are removed, he is given a new name by his torturer: ‘Reek.’ This name not only seems to signify that he is a changed (non-hegemonic masculine) person but also that he is not seen as a person at all. He is named after a descriptor for an unpleasant smell (Gjelsvik and Schubart 2016). As such, Theon Greyjoy’s storyline points to the conclusion that in this fictive phallocentric Game of Thrones world, the only way for men to be (seen as) hegemonic and even to exist as a person in the first place is to have phallic power.

Nonetheless, the two other eunuch characters in *Game of Thrones* display a negotiation with norms of masculinity through which they seem to maintain and/or acquire an alternative position of power that is not only phallocentric. For instance, Lord Varys, who embraces his public persona of pudginess, effeminacy, and powerlessness actually uses his intellectual, social, and manipulating abilities in order to rise to the status of an authority figure: a counselor of the monarch or a puppet master of powerful criminals.

While Varys’ eunuch embodiment proves to be rather insignificant for him in attaining power, Grey Worm’s body plays a more ambiguous role in his quest for power. Grey Worm is an advisor to queen Daenerys and a commander of the Unsullied, an army of soldiers that are castrated and tortured until submissive. Like Varys, Grey Worm places emphasis on his intelligence and his strategic capabilities in leading his army, but his eunuch physique also plays an important role in attaining and maintaining a position of power. One scene wherein Grey Worm duels with one of queen Daenerys’ mercenaries, Daario, clearly exemplifies this. For several hours, Grey Worm and Daario balance their swords on their arms and sit as mirror images opposite to each other. In line with a substantial body of research on symbolic expressions, it may be argued that that their swords figure as substitute penises that potentially provide power (Gade 1989; Mazrui 1974; Mechling 2008; Phelpstead 2007). Then, when queen Daenerys finds Daario and Grey Worm in a duel of symbolic phallic resilience, Daenerys reprimands them for keeping her waiting and tells them that the last man holding his sword must find a new queen (Askey 2018). In response, they simultaneously drop their swords. As such, while their symbolic penis-swords may be interpreted as the focal point of their battle for phallocentric masculine power, dropping their swords may be perceived as symbolizing castration as well as subservience. Interestingly then, while this scene shows that (symbolic) phallocentricity may still be a potential route to power for eunuchs as well as that sword-penises could be perceived as a treat to the establish regime, it also shows that an eunuch may gain and hold power exactly through his (enforced) lack of a (symbolic) phallus. This interpretation is in keeping with records about eunuchs in historical societies – like within Byzantium, Ottoman Turkey and Imperial China – which show that rulers appointed eunuchs to key court positions under the assumption that they would be more likely to serve the good of the empire instead of trying to accrue power for themselves and their families (Scholz 2001; Tougher 2002). In *Game of Thrones*, Grey Worm is indeed presented and reaffirmed as a court-loyal eunuch in dropping his sword, something that secures him of a powerful position close to the ruler.

## The enslaving post-apocalyptic man

Over the last couple of years, infertility is increasingly used as a dystopian trope in fiction television series. *The Handmaid’s Tale* and *The Lottery* are examples of such series. Both series follow the general plotline that men and women are largely infertile which will cause humanity to become extinct in the near future. Given the essential aspect of male infertility in this post-apocalyptic plot, it is striking that the main male characters within these series are portrayed as virile and even heroic hegemonic males. For example, in *The Lottery*, which takes place in a world where no children have been born over the last six years, the male protagonist, Kyle Walker, eventually provides the sperm for one of the hundred artificially manufactured embryos that have to save humankind. The plot of *The Handmaid’s Tale*, moreover, features a totalitarian and theonomic society called Gilead wherein most men and women are infertile, and women are enslaved as handmaids to Gilead’s male commanders for reproductive purposes. Even though the virility of the male protagonist, commander Fred Waterford, is repeatedly questioned, his handmaid Offred (as in: Of Fred) eventually becomes pregnant. In both *The Lottery* and *The Handmaid’s Tale*, then, male infertility remains an abstract given against which post-apocalyptic storylines unfold. That is, infertility seems to be merely used as a tool of narrative suspense and as a building block to when the main male protagonists prove to be fertile up to a point that their virility may save humankind.

However, when focusing on the gender dynamics within *The Handmaid’s Tale*, it becomes clear that the male protagonist’s hegemonic masculinity is profoundly challenged and in need of (re)affirmation beyond his eventually affirmed ability to procreate. As is the case with most post-apocalyptic stories, scarce resources become commodities and are typically allocated within and through totalitarian regimes (Botta 2013; Soncini 2015). In the face of human extinction because of an infertility pandemic, this resource in *The Handmaid’s Tale* is obviously reproductive bodily material and reproductive bodies. In the series, reproductive women are enslaved and allocated to male commanders (and their wives) who repeatedly rape them for the purpose of conceiving. It may be argued that this displayed violence in subordinating, enslaving and raping women not only strongly affirms men’s hegemony over women but also reveals the intensity of the challenge that male infertility – even the threat of it – provides for hegemonic masculinity.

Note that the violent affirmation of Fred’s hegemony gives rise to the question what authority this man depends on in order to enslave women and children. After all, the commodification and allocation of women’s reproductive bodies and materials are not evident. Men also have reproductive material bodies, but they are not put in the position of slaves. Rather, they are represented as slave drivers. In *The Handmaid’s Tale*, women’s enslavement is explicitly legitimized within and through referencing the religious parable of Rachel, Jacob and Leah. This Old Testament story portrays the couple, Jacob and Rachel, who are not able to have children. As the story goes, Rachel suggests that Jacob should have children with her handmaid, Leah. Rachel says: “Behold my maid, Leah. Go unto her and she shall bear upon my knees so that I might also have children by her” (Genesis 30:1). In the series, then, the commanders design the protocol for raping handmaids analogous to this biblical story: Fred rapes and penetrates Offred while she lays – similar to Leah – on the lap of his wife, Serena Joy. All the while, Offred and Serena Joy hold hands. By designing this raping practice analogous to a biblical story, Fred not only ensures an affirmation of his hegemonic position through violence but also attempts to establish and cultivate a tradition of faith surrounding his powerful position. Such a tradition of faith is, according to Ricoeur (2000, 2007), the ultimate recognition and legitimization of power. That is, by mimicking a biblical story within the raping practice, the practice is placed within an established religious tradition of professed hierarchical structures through which the rape itself is not only (attempted to be) legitimized but also Fred’s position as a rapist within this practice.

Besides revealing that a tradition of faith in hegemonic structures may be established through referencing and re-enacting authoritative (religious) traditions and stories, *The Handmaid’s Tale* also shows that the explicit ritual character of this religious reenactment of rape – which is called ‘the ceremony’ in the series – contributes to the sedimentation of this tradition of faith in the professed power structure. Various anthropological studies argue that ritual performances communicate certain traditions of behavior and power structuring as well as hold together those who take part in it by sanctifying that which is communicated within rituals (Kitts 2010; Rappaport 1999; Tambiah 1979). That is, through the sanctification of the message of the ritual, the ritual becomes authoritative and thereby binds its participant together. In doing so, then, the ritual (re-)affirms that which, according to Ricoeur (2000), underlies any (legitimized) power structure: the will to live together as a community. In the case of The Handmaid’s Tale, such sanctification of ritualized rape takes place, quite literally, through the designed similarity of the ‘ceremony’ with a biblical story, and through the prescribed recitation of a biblical text during the rape. Moreover, sanctifying the raping ritual also takes place through the detailed sequence of the steps in the raping ritual, the high degree of formalities involved in it, and the extensive and extraordinary prohibitions and obligations that accompany this ritual. That is, before the rape, Offred should first purify herself by bathing, then there is a communal reading of Scripture, after which Serena Joy is obliged to knock on the door of the room in which the rape will take place. During the rape, all involved persons have a prescribed position, and Fred should not touch Offred apart from penetrating her. Such extraordinary formalities and detailed temporalization give way to a suggestion that the ritual is somehow extramundane (Kitts 2010), ensuring that the behavior and power structures communicated in the ritual become increasingly convincing for those subjected to the ritual. This is also the case for Offred: at the time of the first performance of the ‘ceremony’, she actively questions the practice – asking fellow handmaids, “what I did to deserve this” – but later on in the series, she seems to abide more to the ritual, and eventually defends this practice to others.

## Discussion: diversifying representations of in/fertile men

Infertility is predominantly framed as a female issue in everyday, cultural life. Fiction television series is arguably one of the few cultural expressions in which men’s infertility experiences are represented. Through a content analysis of fiction television series, this study analyzes the portrayal of men who are not able to conceive and undergo a fertility test, of men who consider (not) having a vasectomy, who are involuntarily castrated, and whose alleged infertility threatens the survival of humankind. In taking the concept of hegemonic masculinity as a heuristic tool, I reveal that the common denominator within this array of representations is that these infertile men’s normative, hegemonic masculinity is severely questioned but eventually (almost always) reaffirmed. Infertile men, even when they are displayed as only possibly infertile, are represented as diverging from normative masculinity, that is, from masculine norms of virility, potency, procreation, muscular embodiment, physical toughness, and emotional stoicism. In many cases, this non-/normativity initially excludes these represented men from hegemonic positions. However, given that these representations reveal the extent to which male infertility is (understood as) posing a threat to men’s ability to live up to normative, hegemonic masculinity, almost all men in the analyzed series are eventually able to reaffirm their normativity and acquire hegemonic positions. Notable exceptions to this rule are the characters of Brock in *Reba* and Reek in *Game of Thrones*, whose infertility leads to either an unresolved ambiguous or an absolute undermining of their hegemonic masculinity. Yet except for these two characters, the portrayed infertile men’s hegemony is generally reaffirmed – in line with Connell’s (2005) understanding of acquiring power – within and through their ability to eventually live up to normative masculinity, something which often goes together with coercive force towards women and other men. Such oppression may take on rather subtle forms, for example, by exaggerating the stereotypical female body trait of shortness that invokes powerlessness in order to emphasize the tallness and bodily hegemony of the (possibly) infertile male protagonist (e.g. Marshall in *How I Met Your Mother*). Yet such coercion may also take on more explicit and excessively violent forms, up to a point that one of the series (*The Handmaid’s Tale*) depicts a supposedly infertile man who systematically enslaves and rapes a reproductive woman. Moreover, by drawing on Ricoeur’s (2000) understanding of power structuring, I reveal that some represented men have alternative ways of establishing, defending, and remaining in positions of power. For example, by secretly not having a vasectomy and making their partners pregnant, one of the represented man (Jackson in *Gilmore Girls*) fosters an environment in which his female partner eventually accepts and celebrates her unexpected and male imposed pregnancy. Through such a celebration, she implicitly bestows and confirms what is arguably the precondition for any power structure: the will to continue live together in, in this case, a male hegemonic family (Ricoeur 2000). Even more, some represented men seek and acquire explicit recognition for their normativity and hegemony. By referencing a higher power through reenacting certain biblical stories and/or by designing extramundane rituals within this reenactment, one of the represented men (Fred Waterford in *The Handmaid’s Tale*) appears to succeed in cultivating a tradition of faith that authorizes his power.

This study reveals that representations of men’s infertility experiences in fiction television series all center around (questioning and reaffirming) men’s hegemonic masculinity. As such, these contemporary representations of male infertility present living up to normative, hegemonic masculinity as of vital importance for (possibly) infertile men. Granted, some series depict infertile men in (temporary) subversive positions to other men and women, or portray men who ambiguously relate to certain masculine norms. However, these series also typically portray men as eventually rising to hegemonic positions, or as ultimately discharged from their ambiguous normative masculinity. What is more, in their subservient positions and in their ambiguous non-/normativity, these represented men often still ascribe to and underline the significance of stereotypical hegemonic masculinity for (them as) men, even as human beings. For example, the only represented men who are deemed to be in a permanent subservient position (the eunuch Theon Greyjoy or ‘Reek’ in *Game of Thrones*) are stripped from their humanity within and through their new post-castration names.

While I cannot outline the actual effects on (male) audiences of these representation that univocally signify the vital, even existential importance of hegemonic and normative masculinity traits for in/fertile men, I can point out that such representations may have substantial and potentially harmful repercussions for the ways in which men come to assess and evaluate their infertility and themselves. After all, in capturing cultural narratives about what it (ideally) means to be a man, fiction television series can be understood as the backbone of male identity formation, that is, as the basis for men’s sense-making structures of themselves (Ricoeur 1991). My analysis shows that represented infertile men in television series are generally able to reaffirm their hegemonic masculinity. When confronted with the univocal and existentially disheartening portrayals of in/fertile men in these series, then, male viewers may construct and/or adjust their perceptions of themselves and their lives accordingly. They may endorse perceptions that men are and should be hegemonic males at all times, that living up to normative masculinity is the only way for men to be (considered as) a (hu)man, and that fertility and it adjuvant normative traits is an essential aspect in hegemonic masculinity.

Finally, I would like to make a plea for creating more diverse representations of infertile men in popular culture. Such new representations should entail showing a larger spectrum of how men can experience and deal with their infertility: for example, of infertile men who hold subservient positions that do not deprive them of their humanity or even their masculinity; or better yet, of men whose infertility does not relate to any hierarchical position or norms of masculinity. The point of such a broadened spectrum of representations is to help challenge and transform the current dominant cultural depiction of an in/fertile man as always (wanting to be considered as) normatively male and in (aspiration of) a hegemonic position. Such representations, after all, could show that falling outside of what is regarded as the fertile norm may be part and parcel of life, does not have (significant) repercussions for the ways in which men are considered men and human, and perhaps even that fertility is not a (substantial) normative structuring aspect to begin with. In the end, such representations of non-hegemonic infertile masculinity would not only contribute to a larger array of possible representations that men may draw on in constructing their identity, but would, hopefully, also contribute to a (more) non-hegemonic relationship between men and women.
